# Inter- and intra-observer agreement in the assessment of the cervical transformation zone (TZ) by visual inspection with acetic acid (VIA) and its implications for a screen and treat approach: a reliability study

**DOI:** 10.1186/s12905-022-02131-z

**Published:** 2023-01-19

**Authors:** Khadidja Benkortbi, Rosa Catarino, Ania Wisniak, Bruno Kenfack, Eveline Tincho Foguem, Gino Venegas, Mwanahamuntu Mulindi, Apollinaire Horo, Jose Jeronimo, Pierre Vassilakos, Patrick Petignat

**Affiliations:** 1 Gynecology Division, Department of Pediatrics, Gynecology and Obstetrics, University Hospitals of Geneva, Geneva, Switzerland; 2 Faculty of Medicine, University of Geneva, Geneva, Switzerland; 3 Department of Obstetrics and Gynecology, Faculty of Medicine and Pharmaceutical Science, University of Dschang, Dschang, Cameroon; 4 Gynecology Division, Department of Gynecology and Obstetrics, Clínica Angloamericana, Lima, Peru; 5 Escuela de Medicina Humana, Universidad de Piura, Lima, Peru; 6 University of Zambia, University Teaching Hospital-Women and Newborn Hospital, Lusaka, Zambia; 7 Unit of Gynecology and Obstetrics, University Hospital (CHU) of Yopougon, Abidjan, Côte d’Ivoire; 8 Division of Cancer Epidemiology and Genetics, Department of Health and Human Services, National Cancer Institute, National Institutes of Health, Bethesda, MD 20892 USA; 9 Geneva Foundation for Medical Education and Research, Geneva, Switzerland

**Keywords:** Agreement, Cervical cancer, Visual inspection with acetic acid, Kappa, Transformation zone type, Low-resource setting, International Federation for Cervical Pathology and Colposcopy, Reliability

## Abstract

**Background:**

In low-resource countries, interpretation of the transformation zone (TZ) using the classification of the International Federation for Cervical Pathology and Colposcopy (IFCPC), adopted by the World Health Organization, is critical for determining if visual inspection with acetic acid (VIA) screening and thermal ablation treatment are possible. We aim to assess inter- and intra-observer agreement in TZ interpretation.

**Methods:**

We performed a prospective multi-observer reliability study. One hundred cervical digital images of Human papillomavirus positive women (30–49 years) were consecutively selected from a Cameroonian cervical cancer screening trial. Images of the native cervix and after VIA were obtained. The images were evaluated for the TZ type at two time points (rounds one and two) by five VIA experts from four countries (Côte d’Ivoire, Cameroon, Peru, and Zambia) according to the IFCPC classification (TZ1 = ectocervical fully visible; TZ2 = endocervical fully visible; TZ3 = not fully visible). Intra- and inter-observer agreement were measured by Fleiss’ kappa.

**Results:**

Overall, 37.0% of images were interpreted as TZ1, 36.4% as TZ2, and 26.6% as TZ3. Global inter-observer reliability indicated fair agreement in both rounds (kappa 0.313 and 0.288). The inter-observer agreement was moderate for TZ1 interpretation (0.460), slight for TZ2 (0.153), and fair for TZ3 (0.329). Intra-observer analysis showed fair agreement for two observers (0.356 and 0.345), moderate agreement for two other (0.562 and 0.549), and one with substantial agreement (0.728).

**Conclusion:**

Interpretation of the TZ using the IFCPC classification, adopted by the World Health Organization, is critical for determining if VIA screening and thermal ablation treatment are possible. However, the low inter- and intra-observer agreement suggest that the reliability of the referred classification is limited in the context of VIA. It’s integration in treatment recommendations should be used with caution since TZ3 interpretation could lead to an important referral rate for further evaluation.

*Trial registration* Cantonal Ethics Board of Geneva, Switzerland: N°2017–0110. Cameroonian National Ethics Committee for Human Health Research N°2018/07/1083/CE/CNERSH/SP.

**Supplementary Information:**

The online version contains supplementary material available at 10.1186/s12905-022-02131-z.

## Background

Visual inspection with acetic acid (VIA) is the most affordable method for cervical cancer (CC) screening in low- and middle-income countries (LMICs) [1]**.** The World Health Organization (WHO) recommends visual inspection with acetic acid (VIA) use as triage for HPV-positive women, to provide a single-visit using a “screen, triage and treat” approach [2]. However, since the adoption of VIA in LMICs, significant limitations have been reported, mainly related to its subjectivity and lack of quality control [3, 4].

It is imperative to consider approaches to overcome these concerns and enhance VIA performance. Technical advances in digital imaging allowing enhanced visual assessment by camera or smartphone have been investigated as an adjunct to VIA for the detection of cervical intraepithelial neoplasia grade 2 and worse (CIN2 +) [5, 6]. The adoption of digital imaging in LMICs has been an important step in improving VIA quality control, promoting teaching and image assessment supervision in the context of CC screening [7, 8]. Static digital images have their own limitations because they may not capture dynamic acetowhitening changes; however, their high resolution and the use of a magnified visualisation of the cervix support that digital VIA (D-VIA) may contribute to the identification of CIN2 + [6, 9, 10]. VIA digital images appear to be an acceptable alternative to enhance performance in the absence of colposcopy [10, 11].

Recently, the transformation zone (TZ) classification endorsed by the International Federation for Cervical Pathology and Colposcopy (IFCPC) has been adopted for VIA assessment in LMICs with the aim of improving VIA reporting and treatment [2, 12, 13]. This classification system aims to not only determine the type of TZ, but also the adequate treatment (ablation or excision) [12, 14]. Women screened as HPV-positive should be visually inspected with VIA to determine their eligibility for treatment. Women with a fully visible TZ (TZ type 1 or TZ type 2) upon cervical examination can be safely managed in a single-visit approach with ablative treatment (thermal ablation or cryotherapy), while women with a TZ that is not fully visible (TZ type 3) are considered ineligible for ablation and should be referred for further assessment (requiring, for example, large loop excision of the transformation zone (LLETZ)) [2]. This issue is particularly relevant in LMICs where options for patient referral and LLETZ are limited. This emphasises the need for a reliable classification system that allows accurate differentiation of the TZ type [2].

## Methods

### Aim, design and setting of the study

We aimed to evaluate the reliability of TZ type assessment using the IFCPC classification [13] applied on digital VIA cervical images obtained from a CC screening campaign. We performed a multi-observer reliability study using prospectively collected data. Inter- and intra-observer agreement of TZ classification were measured among 5 experts assessing the digital images. This study is part of a CC screening trial termed the 3 T-Approach (for Test-Triage-Treat on the same day), which has been conducted since 2018 in the West Region of Cameroon [13]. Briefly, women with an HPV-positive screening test were invited for a pelvic exam and cervical visual assessment. Cervical images of the native cervix and following application of acetic acid (1 min after application) were taken. Of the 380 HPV-positive women screened between March and October 2019, the first 100 consecutive cases fulfilling the criteria for this ancillary study were included.

### Characteristics of participants and description of materials

Inclusion criteria of patients were volunteer women aged between 30 and 49 years old, recruited at the cervical cancer screening campaign at the Dschang District Hospital (West Region of Cameroon), with an HPV-positive diagnosis at the initial screening visit. Cervical images were collected from initial and follow-up visits during which a VIA examination was performed. Exclusion criteria were those of the main trial (pregnancy, previous history of cervical surgery, or hysterectomy). Additionally, poor-quality images (blurry, incomplete cervix exposure, bleeding, or cervix covered by thick cervicovaginal mucus) were also excluded by the study investigators (KB and PP) prior to the expert assessment.

### Cervical image capture and selection

Images were captured with a smartphone (Samsung Galaxy S5), which has a 16-megapixel camera. The flash mode (LED) was permanently activated using a mobile health application named “Cervical Cancer Prevention System” for digitalized CC screening monitoring [15, 16]. Images were taken at an approximately 1 cm distance from the speculum using a universal digital camera support and bracket, where the smartphone was easily adjusted [15]. Cervical images were captured for consecutive participants with good quality images and no other selection criteria, corresponding to “real life conditions” of TZ prevalence.

### Description of process

Five international VIA experts were invited to interpret 100 consecutive digital images uploaded onto an online database (www.jotform.com).

The observers were aware that the digital images were provided in a successive order and observers were asked to define the type of TZ according to the IFCPC classification. The IFCPC defines a fully visible and ectocervical TZ as type 1 (TZ1), a partially or completely endocervical TZ that is fully visible as type 2 (TZ2), and a partially or completely endocervical TZ that is not fully visible as type 3 (TZ3) [13]. The experts independently reviewed 100 cases each (native and after acetic acid application) [12, 14].

The online survey was performed at two different time points at a 2–3-month interval (images were provided in reverse order the second time). No feedback was given to the investigators between the two rounds.

### Methodological quality assessment

An online evaluation form explained the study procedure. To support the observers in their understanding of the classification used, an original description and schematic illustration of TZ were available on the platform [13]. Observers evaluated the photo quality and whether it was of sufficient quality for TZ interpretation. If more than two observers considered that the image quality was insufficient in both rounds, it was excluded from statistical analysis. Inter- and intra-observer agreement for TZ interpretation was assessed.

### Statistical analysis

Agreement between the colposcopy experts was measured using the kappa statistic (ĸ). Based on Temel and Erdogan’s sample size tables in agreement studies, we estimated that a sample size of 96 cervical images would be sufficient to provide an 80% power at an alpha level of 0.05 to estimate the kappa coefficient, if the ĸ is at least 50%. Quantitative variables are expressed as means and standard deviations, and qualitative variables are expressed as percentages, unless otherwise stated. The statistical method used to compare the intra- and inter-observer agreement of the TZ type was Fleiss’ kappa. The following values were considered: 1.00–0.81, almost perfect agreement; 0.80–0.61, substantial agreement; 0.60–0.41, moderate agreement; 0.40–0.21, fair agreement; 0.20–0.00, slight agreement; and < 0, disagreement. A secondary analysis of inter-observer agreement was conducted, defining a “consensus diagnosis” as at least three observers agreeing during both the first and second rounds. For instance, three or more observers had to agree on the TZ type to consider their answer as the reference standard. If an image did not achieve a majority agreement (i.e., only one or two observers agreed on each TZ type), it was excluded from this secondary inter-observer agreement analysis. The proportion of each TZ type selected by observers was calculated. We also explored the agreement variation when TZ1 was classified together with TZ2 versus TZ3, as well as TZ2 and TZ3 together versus TZ1 (binary answer). The data were analysed using a statistical analysis software package (statacorp.2013. Stata Statistical Software: Release 13. College Station, TX, USA).

## Results

### Socio-demographic characteristics

The average age of the experts (four men and one woman) was 52.6 ± 4.2 years. All are recognized gynaecologists with a specialization in gynaecologic oncology or in the screening and prevention of cervical cancer and counting between 8 and 28 years of experience in VIA, with or without experience in colposcopy. They reported having performed more than 300 VIA and had a median of 15 years of VIA experience in different LMICs, including Peru (n = 2), Cameroon (n = 1), Côte d’Ivoire (n = 1), and Zambia (n = 1) (Table 1). The median age of participants who provided cervical images was 40 years old (IQR 33–43), among whom 5% were HIV positive. The median age of first intercourse was 18 (IQR 14–25), with a median of 3 sexual partners (IQR 1–20). Most used no contraception (64%). Only one woman was nulligravida and 3 were nulliparous.

**Table 1:** Observers' sociodemographic characteristics

Variable	N (%)
Total	5 (100)
Age (years), mean (± SD)	52.6 ± 4.2
*Sex*	
Male	4 (80.0)
Female	1 (20.0)
Years of experience in cervical cancer prevention, median (IQR)	15 (10)
*Number of visual inspections with acetic acid during lifetime*	
> 300	5 (100)
*Number of visual inspections per year*	
1–30	0
31–60	1 (20.0)
> 60	4 (80.0)

*SD* standard deviation;* IQR* interquartile range

#### Assessment of image quality

Most of the images were considered of good quality by the reviewers in the first (78%- 86% of good quality images among the five different experts) and second rounds (82%-99%). No image was classified as insufficient for TZ interpretation by more than two observers; therefore, no case was excluded (Table 2). The proportion of good quality images improved in the second round for all experts except for observer II. However, the difference in the mean proportion of images considered of good quality between both evaluation rounds did not reach statistical significance (81.8% in the first round vs. 89.4% in the second round, p = 0.100).

**Table 2:** Images considered of good quality for diagnosis

Observers	First round (%)	Second round (%)
I	78	90
II	86	83
III	86	93
IV	78	82
V	81	99

### Assessment of TZ classification

In the first round, the overall kappa value in TZ interpretation was moderate for TZ1 (0.460), slight for TZ2 (0.153), and fair for TZ3 (0.329) (Table 3).

**Table 3:** Overall kappa values for the transformation zones

TZ	Kappa—first round	Kappa—second round
1	0.460	0.343
2	0.153	0.171
3	0.329	0.341

Observers mainly disagreed on TZ2 classification. Overall, 37.0% of VIA images were interpreted as TZ1, 36.4% as TZ2, and 26.6% as TZ3. The second round showed a different proportion of TZ1 and TZ2, at 44.2% and 29.4%, respectively. The TZ3 proportion in the second round was similar at 26.4% (Table 4).

**Table 4:** Transformation zone classification by observers

TZ	Observers—First round					
	I (n %)	II (n %)	III (n %)	IV (n %)	V (n %)	All n (%)
1	34	46	30	36	39	185 (37.0)
2	38	40	34	35	35	182 (36.4)
3	28	14	36	29	26	133 (26.6)

TZ	Observers—Second round					
	I (n %)	II (n %)	III (n %)	IV (n %)	V (n %)	All n (%)
1	30	55	39	51	46	221 (44.2)
2	40	30	40	20	17	147 (29.4)
3	30	15	21	29	37	132 (26.4)

### Inter-observer agreement

Among the 100 images, 19% of images had perfect inter-observer agreement in both rounds (round 1: 14% TZ1, 1% TZ2, 4% TZ3 vs. round 2: 15% TZ1, 0% TZ2, 4% TZ3). There was almost perfect inter-observer agreement (at least four out of five observers agreed) for 47% of the images in the first round (24% TZ1, 12% TZ2, 11% TZ3) and 47% in the second round (27% TZ1, 9% TZ2, 11% TZ3). The global agreement between all experts was fair in both rounds, with a kappa of 0.313 (95% CI 0.261–0.353) in the first round and 0.288 (95% CI 0.240–0.329) in the second round.

Inter-observer agreement between two observers ranged from slight to moderate (kappa 0.102–0.531) in the first round and from slight to fair (kappa 0.180–0.391) in the second round. The strongest agreement was between observers I and V in the first round (moderate; kappa 0.531) but was not consistent in the second round (fair; kappa 0.385).

**Fig. 1:**
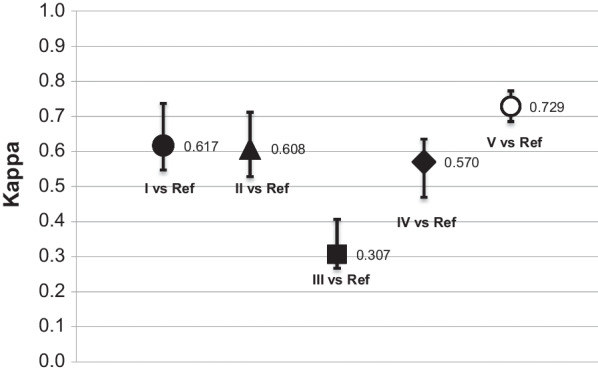
Inter-observer agreement for TZ classification compared with the reference standard (Fleiss’ kappa and 95% CI): First round. Reference (Ref) : TZ according to the majority of responses from the participants. For instance, when three or more participants considered an image to be TZ2, ref was TZ2. If there was a tie (two participants considered the image to be TZ2 and two participants to be T3), this image was excluded from the analysis

Figure 1 demonstrates the inter-observer agreement related to each observer compared with the reference standard (“consensus diagnosis”) in the first round. Three experts reached substantial agreement (kappa 0.729, 0.617, and 0.608) and one achieved moderate agreement (kappa 0.570). Observer III had fair agreement (kappa 0.307).

#### Intra-observer agreement

Observer I showed substantial intra-observer agreement (kappa 0.728), while observers IV and V had moderate intra-observer agreement (kappa 0.562 and 0.549), and observers II and III had fair intra-observer agreement (kappa 0.356 and 0.345) (Table 5).

**Table 5:** Inter- and intra-observer agreement for TZ classification: First and second round

	Observer								
	I	II (R1)	II (R2)	III (R1)	III (R2)	IV (R1)	IV (R2)	V (R1)	V (R2)
I	0.728* (0.654–0.775)	**0.418** (0.294–0.503)	**0.284 (**0.190–0.406)	**0.102** (0.062–0.167)	**0.227** (0.189–0.338)	**0.382** (0.328–0.470)	**0.382** (0.323–0.444)	**0.531** (0.476–0.586)	**0.385** (0.287–0.405)
II	_	0.356* (0.269–0.378)		**0.112** (− 0.050–0.177)	**0.180** (0.138–0.208)	**0.251** (0.165–0.330)	**0.367** (0.323–0.461)	**0.410** (0.357–0.520)	**0.219** (0.181–0.226)
III	–	–		0.345* (0.187–0.433)		**0.222** (0.173–0.272)	**0.197** (0.188–0.274)	**0.269** (0.213–0.346)	**0.304** (0.206–0.346)
IV	–	–		–		0.562* (0.450–0.719)		**0.471** (0.379–0.649)	**0.391** (0.267–0.476)
V	–	–		–		–		0.549* (0.535–0.623)	

Values are Fleiss’ kappa (95% CI)  for inter- and intra-observer agreement

*Intra-observer agreement was calculated from the assessments given in the first and second rounds by the same participant

CI Confidence interval; R1: TZ interpretation in the first round; R2: TZ interpretation in the second round. The five experts are referred as I, II, III, IV, V

### Assessment of coupled TZ classification

When TZ1 and TZ2 were coupled, the inter-observer agreement ranged from kappa 0.042 to 0.594 in the first round and kappa 0.089 to 0.639 in the second round, and the intra-observer agreement ranged from kappa 0.234 to 0.806 (median kappa 0.566). When the TZ2 was coupled with TZ3, inter-observer agreement ranged from kappa 0.256 to 0.677 in the first round and kappa 0.178 to 0.504 in the second round, and intra-observer agreement ranged from kappa 0.452 to 0.771 with a higher median agreement (median kappa 0.662). For more detailed agreement results see Additional file 1.

## Discussion

### Main findings

Our findings support that there was large variability in TZ assessment performed by different VIA experts, with fair inter-observer agreement in both rounds (kappa 0.313 and 0.288). TZ classification in clinical practice appears to be a method associated with low reliability and large variation in its interpretation. This suggests that TZ assessment is challenging to interpreted and reproduced, with TZ2 showing the highest heterogeneity.

### Interpretation

Vallikad et al. [17] reported in a colposcopy context among three reviewers [17], reported higher inter-observer (kappa 0.53–0.66) and intra-observer (kappa 0.60–0.86) agreement for TZ type classification than in our study, but like our findings, the lowest agreement between observers was found for TZ2 [17, 18].

In real-life conditions, the manipulation of the cervix to differentiate TZ2 from TZ3 might reduce TZ2 heterogeneity. An exploratory analysis was therefore performed combining images classified as [TZ2 or TZ3] versus TZ1 (Additional file 1). Our results showed that inter- and intra-observer agreement were improved by combining TZ2 with TZ3. In contrast, TZ1 being fully ectocervical, its interpretation is not expected to depend on cervical manipulation. Nevertheless, combining TZ1 with TZ2 also showed improved intra- and inter-observer agreement, suggesting that the increase in agreement in both cases of combined TZ types may be in part due to the lower number of categories being compared. Furthermore, despite the improved Kappa after combining TZ2 and TZ3, overall agreement remains relatively low (only 10% of inter-observer comparisons showing substantial agreement across both rounds), supporting the hypothesis that even in real-life conditions, the level of heterogeneity in the interpretation of TZ remains significant. Further studies should confirm this by assessing TZ agreement based on on-site interpretation with the possibility to manipulate the cervix.

The IFCPC TZ classification was primarily developed to improve colposcopy reporting and to define the type of excisional therapy (generally LLETZ) indicated in cases of precancerous lesions [12]. Current diagnostic procedures of colposcopy in high-income countries are cervical biopsy in cases of TZ1 or TZ2 and endocervical curettage (ECC) for TZ3 to obtain fragments of squamous epithelium from inside the cervical canal. However, in low-resource settings, these procedures (colposcopy, biopsy, LLETZ) are not readily available most of the time and are not feasible in a “screen-and-treat” approach, requiring a multi-visit approach with referral for further evaluation.

Reducing the number of clinical visits is a strategy recommended by the WHO in LMICs because it increases compliance and follow-up while reducing program costs [2]. In this context, the endorsed TZ classification by the WHO should help clinicians to determine which patients can be safely evaluated with VIA and treated by ablation, and those who are inadequately evaluated by VIA and require referral for additional management [2, 19]. The TZ3 prevalence observed in our population was 26.6% (Table 4, first round), indicating that a significant number of women may require referral and additional investigation. In the literature from high-income countries, a great variation in TZ3 prevalence was reported, ranging between 16.3 and 80% [17–19]. In low-resource contexts, the front-line provider’s decision to refer women with TZ3, has important consequences for both the women and the health care system, with notable impacts on logistics and service delivery, as this requires additional time, equipment, financial resources, and transportation.

### Strengths and limitations

The main limitation of this study is that the observers were aware that their interpretations were not used for clinical decision-making; therefore, the results may not fully reflect real-life practice.

There are also some strengths to highlight. In this study, the cases were not selected other than for image quality. Images were presented in a consecutive order, with cases corresponding to a real-life distribution of TZ types in a routine screening setting. Furthermore, the TZ interpretation was performed by international experts with extensive experience in VIA.

### Practical and research recommendations

Considering the heterogeneity of TZ interpretation and its consequences on patient management, the importance of long-term follow-up of HPV-positive patients should be emphasized to make up for potentially missed diagnoses or inappropriate treatment. These considerations should be integrated in the initial and continuous training of health care providers practicing VIA and treatment of precancerous cervical lesions.

Further investigations to optimise the management of TZ3 in low-resource contexts as well as to reduce the variability in TZ3 interpretation should be explored. Investigation of surrogate markers that may help to stratify the risk of HPV-positive women with TZ3 and determine who can be safely offered conservative management should be pursued. In addition, recent development of an artificial intelligence algorithm might assist front-line providers, not only in the detection of precancerous lesions, but also to define participants who are eligible for treatment [20].

## Conclusion

The variability in inter- and intra-observer agreement between VIA specialists suggests that the reliability of TZ interpretation is limited in the context of VIA and therefore it’s integration in treatment recommendations should be used with caution. Implementation of TZ interpretation in resource-limited contexts has important logistical and operational implications because a significant number of participants with TZ3 require a multi-visit approach due to referral for further evaluation.

## Supplementary Information

**Additional file 1.** Intra- and inter-observer agreement for separate and coupled TZ classification.

## Data Availability

The datasets used and/or analyzed during the current study are available from the corresponding author on reasonable request. In accordance with the journal’s guidelines, we will provide our data for the reproducibility of this study if such is requested.

## Abbreviations

TZ: Transformation zone
TZ1: Transformation zone type 1
TZ2: Transformation zone type 2
TZ3: Transformation zone type 3
IFCPC: International Federation for Cervical Pathology and Colposcopy
VIA: Visual inspection with acetic acid
D-VIA: Digital visual inspection with acetic acid
CC: Cervical cancer
LMICs: Low- and middle-income countries
WHO: The World Health Organization
HPV: Human papillomavirus
CIN2 +: Cervical intraepithelial neoplasia grade 2 and worse
LLETZ: Large loop excision of the transformation zone
3 T-Approach: Test-triage-treat on the same day
