# Supplementary material for: Inter- and intra-observer agreement in the assessment of the cervical transformation zone (TZ) by visual inspection with acetic acid (VIA) and its implications for a screen and treat approach: a reliability study
Source: BMC Womens Health. 2023 Jan 19;23:27. doi: 10.1186/s12905-022-02131-z (PMC9854065; doi:10.1186/s12905-022-02131-z)
Supplement: Supplementary file 1 — Additional file 1. Intra- and inter-observer agreement for separate and coupled TZ classification. [file 12905_2022_2131_MOESM1_ESM.docx]

**Supplementary file**

**Supplementary file 1:** Intra- and inter-observer agreement for separate and coupled TZ classification.

|  | **First round** | | | | | | |  |
| --- | --- | --- | --- | --- | --- | --- | --- | --- |
|  | TZ1. TZ2. TZ3 | |  | TZ1–2. TZ3 | |  | TZ1. TZ2–3 | |
|  | Kappa (95% CI) | | | | | | |  |
| I vs. II | 0.418^C^ | (0.355–0.430) |  | 0.415^C^ | (0.410–0.537) |  | 0.589^C^ | (0.511–0.608) |
| I vs. III | 0.102^A^ | (−0.012–0.170) |  | 0.042^A^ | (−0.043–0.104) |  | 0.312^B^ | (0.091–0.374) |
| I vs. IV | 0.382^B^ | (0.311–0.499) |  | 0.534^C^ | (0.494–0.588) |  | 0.429^C^ | (0.356–0.526) |
| I vs. V | 0.531^C^ | (0.491–0.561) |  | 0.594^C^ | (0.485–0.632) |  | 0.677^D^ | (0.569–0.733) |
| II vs. III | 0.112^A^ | (0.025–0.145) |  | 0.098^A^ | (−0.043–0.209) |  | 0.256^B^ | (0.137–0.295) |
| II vs. IV | 0.251^B^ | (0.200–0.310) |  | 0.341^B^ | (0.083–0.600) |  | 0.427^C^ | (0.363–0.555) |
| II vs. V | 0.410^C^ | (0.298–0.479) |  | 0.389^B^ | (0.362–0.474) |  | 0.613^D^ | (0.559–0.700) |
| III vs. IV | 0.222^B^ | (0.167–0.300) |  | 0.161^A^ | (0.043–0.273) |  | 0.324^B^ | (0.169–0.440) |
| III vs. V | 0.269^B^ | (0.029–0.356) |  | 0.261^B^ | (0.127–0.412) |  | 0.408^C^ | (0.324–0.451) |
| VI vs. V | 0.471^C^ | (0.351–0.588) |  | 0.574^C^ | (0.439–0.680) |  | 0.552^C^ | (0.444–0.698) |
|  |  |  |  |  |  |  |  |  |
|  | **Second round** | | | | | | | |
|  | TZ1. TZ2. TZ3 | |  | TZ1–2. TZ3 | |  | TZ1. TZ2–3 | |
|  | Kappa (95% CI) | | | | | | | |
| I* | 0.728^D^ | (0.650–0.730) |  | 0.806^D^ | (0.726–0.851) |  | 0.771^D^ | (0.716–0.859) |
| II* | 0.356^B^ | (0.272–0.414) |  | 0.234^B^ | (0.155–0.411) |  | 0.504^C^ | (0.456–0.539) |
| III* | 0.345^B^ | (0.297–0.349) |  | 0.451^C^ | (0.398–0.483) |  | 0.452^C^ | (0.352–0.466) |
| IV* | 0.562^C^ | (0.486–0.627) |  | 0.660^D^ | (0.315–0.677) |  | 0.662^D^ | (0.632–0.808) |
| V* | 0.549^C^ | (0.448–0.609) |  | 0.566^C^ | (0.391–0.736) |  | 0.695^D^ | (0.523–0.796) |
| Median | 0.549^C^ |  |  | 0.556^C^ |  |  | 0.662^D^ |  |
| I vs. II | 0.284^B^ | (0.190–0.406) |  | 0.472^C^ | (0.282–0.662) |  | 0.327 | (0.273–0.423) |
| I vs. III | 0.227^B^ | (0.189–0.338) |  | 0.089^A^ | (−0.110–0.288) |  | 0.276^B^ | (0.131–0.327) |
| I vs. IV | 0.382^B^ | (0.323–0.444) |  | 0.639^D^ | (0.473–0.805) |  | 0.385^B^ | (0.320–0.454) |
| I vs. V | 0.385^B^ | (0.287–0.405) |  | 0.442^C^ | (0.259–0.625) |  | 0.504^C^ | (0.363–0.645) |
| II vs. III | 0.180^A^ | (0.138–0.208) |  | 0.125^A^ | (−0.092–0.341) |  | 0.178^A^ | (0110–0.399) |
| II vs IV | 0.367^B^ | (0.323–0.461) |  | 0.490^C^ | (0.299–0.682) |  | 0.439^C^ | (0.339–0.492) |
| II vs. V | 0.219^B^ | (0.181–0.226) |  | 0.169^A^ | (−0.007–0.345) |  | 0.385^B^ | (0.321–0.488) |
| III vs. IV | 0.197^A^ | (0.188–0.274) |  | 0.154^A^ | (−0.051–0.358) |  | 0.204^B^ | (0.109–0.269) |
| III vs. V | 0.304^B^ | (0.206–0.346) |  | 0.293^B^ | (0.107–0.480) |  | 0.369^B^ | (0.275–0.406) |
| VI vs. V | 0.391^B^ | (0.267–0.476) |  | 0.506^C^ | (0.330–0.683) |  | 0.421^C^ | (0.222–0.665) |

Intra*- and inter-observer agreement for TZ classification: First and second rounds according to three different groups (TZ1 vs. TZ2 vs. TZ3; TZ1+2 vs. TZ3; TZ1 vs. TZ2+3)

| ^A^ | Slight (kappa 0.01–0.20) |
| --- | --- |
| ^B^ | Fair (kappa 0.21–0.40) |
| ^C^ | Moderate (kappa 0.41–0.60) |
| ^D^ | Substantial (kappa 0.61–0.80) |
